# Extracts of *Argemone mexicana* L. Contain Antifungal Compounds for the In Vitro Control of *Monilinia fructicola*, *Colletotrichum gloeosporioides, Fusarium oxysporum,* and *Sclerotinia sclerotiorum*: Preliminary Evidence for Field Application

**DOI:** 10.3390/biotech14040082

**Published:** 2025-10-26

**Authors:** Iridiam Hernández-Soto, Antonio Juárez-Maldonado, Alfredo Madariaga-Navarrete, Ashutosh Sharma, Antonio de Jesus Cenobio-Galindo, Jose Manuel Pinedo-Espinoza, Aracely Hernández-Pérez, Alma Delia Hernández-Fuentes

**Affiliations:** 1 Instituto de Ciencias Agropecuarias, Universidad Autónoma del Estado de Hidalgo, Av. Universidad Km. 1, Rancho Universitario, Tulancingo de Bravo 43600, Hidalgo, Mexico; iridiam_hernandez@uaeh.edu.mx (I.H.-S.); alfredo_madariaga@uaeh.edu.mx (A.M.-N.); antonio_cenobio@uaeh.edu.mx (A.d.J.C.-G.); he409779@uaeh.edu.mx (A.H.-P.); 2 Departamento de Botánica, Universidad Autónoma Agraria Antonio Narro, Saltillo 25315, Coahuila, Mexico; antonio.juarez@uaaan.edu.mx; 3 Centro de Bioingeniería, Escuela de Ingeniería y Ciencias, Tecnológico de Monterrey Campus Querétaro, Av. Epigmenio González, Fracc., San Pablo 76130, Querétaro, Mexico; asharma@tec.mx; 4 Unidad Académica de Agronomía, Universidad Autónoma de Zacatecas, km 15.5 Carretera Zacatecas-Guadalajara, Zacatecas 98170, Zacatecas, Mexico; pinedozac_uaa@uaz.edu.mx

**Keywords:** sustainability, plant extracts, organic farming, phytopathogens, secondary metabolites

## Abstract

*Argemone mexicana* L. is considered a weed; however, it contains secondary metabolites that can control phytopathogenic fungi in vitro, with the potential to adapt its effectiveness in the field. In the present study, leaf extracts of *A. mexicana* (hexane and methanol) were prepared, and their chemical profiles were analyzed using gas chromatography–mass spectrometry (GC-MS). The in vitro antifungal activity of each extract was evaluated at different concentrations (500, 1000, 2000, 4000, and 8000 mg L^−1^) against phytopathogens such as *Monilinia fructicola, Colletotrichum gloeosporioides, Fusarium oxysporum*, and *Sclerotinia sclerotiorum*. Based on their chemical profiles, 14 compounds were identified in the hexanic extract, and 11 compounds were identified in the methanolic extract. These compounds included those with antifungal activity, such as Benzene; 1.3-bis(1.1-dimethylethyl)-; pentanoic acid; 5-hydroxy-, 2,4-di-1-butylphenyl esters; 1,2,4-Triazol-4-amine; and N-(2-thienylmethyl). The hexanic extract demonstrated fungistatic activity on the four fungi tested, while the methanolic extract exhibited fungicidal activity against *C. gloeosporioides* and *F. oxysporum*. The results of the Probit analysis showed variations in the sensitivity of phytopathogenic fungi to the treatments evaluated. In *M. fructicola*, the hexane extract presented an EC_50_ of 317,146 mg L^−1^ and an EC_90_ of 400,796 mg L^−1^. For *C. gloeosporioides*, the EC_50_ was 2676 mg L^−1^ and the EC_90_ was 888,177 mg L^−1^, while in *F. oxysporum* an EC_50_ of 34,274 mg L^−1^ and an EC_90_ of 1528 mg L^−1^ were estimated. In the case of *S. sclerotiorum*, an EC_50_ of 560 mg L^−1^ and an EC_90_ of 7776 mg L^−1^ were obtained. Finally, for the commercial fungicide Captan^®^, an EC_50_ of 1.19 mg L^−1^ and an EC_90_ of 1.67 mg L^−1^. These results suggest that extracts from *A. mexicana* could provide a natural alternative for the control of phytopathogenic fungi.

## 1. Introduction

Plant diseases caused by phytopathogenic fungi are responsible for major economic losses, such as lost crop yields, due to issues with product quality and safety. The most common phytopathogenic fungi include *Monilinia fructicola* (G. Wint.) Honey, *Colletotrichum gloeosporioides* (Penz.), *Fusarium oxysporum* Schltdl., and *Sclerotinia sclerotiorum* (Lib. de Bary) [1]. *Monilinia fructicola* is an ascomycetous fungus that causes brown rot in many stone fruits [2]. Most of the damage occurs postharvest, resulting in yield losses of up to 80% [3]. *Colletotrichum gloeosporioides* is the causative agent of anthracnose, a disease that affects a wide range of horticultural and fruit crops. Damage can occur at any stage of the production chain: field growth, storage, and postharvest marketing [4,5]. Anthracnose symptoms include cuticular and subcuticular lesions on the stem, leaves, and inflorescence, with the growth destructive of tissue (necrosed tissue) [6]. Fruit lesions on the epidermis begin as small circles that enlarge and darken. In advanced stages, the fungus produces acervuli containing numerous conidia ranging from white to salmon in color [7]. *Fusarium oxysporum* causes destructive vascular wilt and root rot [8]. This fungus penetrates through specialized hyphae and colonizes the cortex through intracellular and intercellular growth [9]. *Sclerotinia sclerotiorum* is a homothetic necrotrophic plant pathogen that is responsible for cotton soft rot [10]. This fungus infects more than 400 plant species worldwide, including important crops such as sunflower, oilseed rape, soybean, bean, pea, lentil, chickpea, potato, lettuce, carrot, cabbage, celery, pepper, and poppy seeds [11].

Plant protection against these phytopathogens is a primary concern for the agricultural sector. Since their initial appearance, some of these devastating diseases have been controlled using synthetic fungicides [12]. However, the inappropriate use of fungicides in agriculture has a negative impact on the environment and human health, resulting in the development of fungicide-resistant fungi [13]. New ecological alternatives are leading to a reduction in synthetic fungicides [14]. Extracts from wild plants are an excellent resource for obtaining new natural fungicides due to their content of secondary metabolites [15]. In addition, these extracts are considered environmentally friendly agents because of their natural origins and typically have limited field persistence and a shorter shelf life compared to synthetic fungicides. Overall, they have the potential to reduce the accumulation of persistent residues and environmental pressure. However, some naturally occurring compounds can also generate undesirable metabolites or residues, so their safety and stability must be assessed [16,17]. These biofungicides utilize several mechanisms of action against phytopathogens, including inhibition of germ tube elongation, delay of sporulation, DNA damage, inhibition of protein synthesis, and damage to hyphal and mycelial structures [18].

An ecological alternative for the control of diseases caused by phytopathogenic fungi is *Argemone mexicana* L., also known as chicalote, a species endemic to Mexico and widely distributed throughout the continent. It is generally considered a weed but contains alkaloids, terpenoids, flavonoids, phenolic compounds, long-chain aliphatic compounds, and some aromatic compounds that are thought to have antifungal activity [19]. For example, Singh et al. [20] evaluated the methanolic extract of chicalote for antifungal activity against *Ustilago cynodontis* (Pass.) Henn., *Cercospora cajani* Henn, and *Sphaerotheca* spp. In their study, fungicidal activity was attributed to compounds such as caffeic acid, ferulic acid, and tannic acid. Another study [21] assessed the fungicidal activity of an aqueous extract and ethanolic extract of *A. mexicana* against *Botrytis cinerea* and *Cladosporium* spp. Resistant activity was attributed to the compounds carvacrol, 7,9-Di-tert-butyl-1-oxaspiro[4.5]deca-6,9-diene-2,8-dione, allocryptopine, and oxyberberine. Carvacrol, in particular, has been shown to negatively affect the permeability of cell membranes and cause a marked decrease in the total lipid content of cells in phytopathogenic fungi, suggesting the destruction of cell membrane structures [22], while allocryptopine exhibits cytotoxic and antimicrobial effects that compromise the structural integrity of pathogenic fungi [23].

However, most studies reported to date have been limited to insufficient phytopathogen numbers and have not examined the effect of solvent polarity on the extraction of bioactive compounds from *A. mexicana*. Therefore, the present study provides novel evidence by evaluating the antifungal activity of *A. mexicana* extracts against the phytopathogenic fungi *M. fructicola*, *C. gloeosporioides*, *F. oxysporum*, and *S. sclerotiorum*. By using two solvents with different polarities, hexane (non-polar) and methanol (polar), we aimed to extract the greatest possible diversity of secondary metabolites [18]. The extracts were analyzed using gas chromatography coupled with mass spectrometry (GC-MS), an analytical tool used for the qualitative analysis of plant matrices due to its high sensitivity and selectivity for volatile and semi-volatile compounds [24]. We hypothesize that the polarity of the solvent modifies the chemical profile of the extracts and, consequently, their antifungal efficacy.

## 2. Materials and Methods

### 2.1. Plant Material

**Figure 1:**
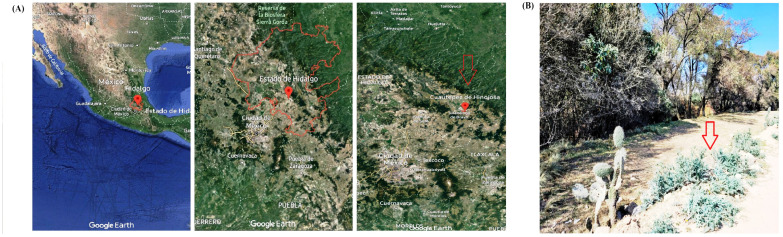
(**A**) *Argemone mexicana* L. plant material collection site; (**B**) *Argemone mexicana* L. plants in vegetative development, the red arrow indicates the species used for the experiment. Map data ©2025 Google.

The leaves of *A. mexicana* L. were randomly collected at the vegetative development stage during winter in the region of Cuautepec de Hinojosa, Hidalgo, Mexico, at 20° 09′00″ N, 98′00″ W, and an altitude of 2200–2900 m above sea level. This region has a cold climate with abundant vegetation, natural resources, and semi-arid soils rich in organic matter and nutrients (Figure 1). The identification of species was carried out in the Botany Laboratory of the Institute of Biological Sciences of the Autonomous University of the State of Hidalgo. Leaves were stored at −70 °C (Thermo Scientific 703 Ultra-Low Freezer, Grand Island, NY, USA) and then preserved in a freeze-dryer (Model 79480; Labconco Corporation, Kansas City, MO, USA). The leaves were then ground in a blade grinder (GM 200, Grindomix, Glen Mills Inc., Clifton, NJ, USA) at 10,000 rpm for one minute, and the samples were stored for later use in a desiccator at 26 °C and 0% humidity.

### 2.2. Extraction of the Phytochemical Compounds

The plant extracts were obtained by mixing previously freeze-dried plant material with either hexane or methanol. Each solvent (500 mL) was mixed with 50 g of plant material, and the mixtures were soaked for 15 days. Then, the extracts were filtered twice through Whatman filter paper No. 1. The solvents of the extracts were removed under vacuum using a rotary evaporator (BUCHI model R-215, Equipar, Diclab™,Zapopan, Jalisco, Mexico) for four hours at a temperature of 40 °C and a pressure of 100 mbar for hexane and 60 mbar for methanol, as indicated by the device. We followed the methodology proposed by De Rodríguez [25] with some modifications, specifically in the amount of sample used. The extracts were stored in a desiccator at 26 °C and 0% humidity until use in the bioassays. This procedure was repeated throughout the experiment to ensure a sufficient supply of plant extracts for the various microbiological assays. On average, the extract yield was 44% for hexane and 68% for methanol (22 and 34 g of solids, respectively).

### 2.3. Gas Chromatography-Mass Spectrometry Analysis (GC-MS)

Data were collected as previously described by Hernández-Soto et al. [26]. For analysis, 1 g of each dried extract was dissolved in 20 mL of either hexane or methanol. The resulting solutions were filtered through Whatman No. 1 filter paper to remove solid particles and ensure clarity, conforming to the methodology in [27]. This procedure was repeated four times with three replicates per treatment. Specifically, the chromatographic analysis was performed on an Agilent Technologies 7890A GC system, Santa Clara CA, USA connected to a 5975 GC/MSD in splitless scan mode. Metabolite separation of the extracts was performed on a DB17HT column (30 m × 0.25 mm 1D × 0.15 µL) and an ionization system with energy at 70 eV. Helium was the carrier gas with a constant flow of 3 mL/min. A sample volume of 1 µL was injected at a temperature of 270 °C. Data were analyzed using Agilent MassHunter (B.07) software and AMDIS32 V2.1. The mass spectra of the compounds were analyzed using the National Institute of Standards and Technology (NIST) database, considering only those compounds present in at least 70% of the runs and with a minimum similarity index of 800 (Match Factor). We also identified their biological plausibility and bibliographic support, according to the methodology proposed in [28].

### 2.4. In Vitro Evaluation of the Extracts Against Phytopathogenic Fungi

The strains used in this study were donated by the Agricultural and Environmental Chemistry Laboratory of the Autonomous University of the State of Hidalgo. They have the following Genbank accession numbers: *Monilia fructicola*: MN179292; *Colletotrichum gloeosporiodes*: MT850050; *Fusarium oxysporum*: MK605264; and *Sclerotinia sclerotiorum*: ON401416. The antifungal activity of *A. mexicana* leaf extracts on phytopathogenic fungi was evaluated using the agar dilution method with some modifications, specifically for the concentrations of the extract evaluated [29]. The various extracts were incorporated into the Potato Dextrose Agar (PDA) culture medium after sterilization at 121 °C for 15 min in an autoclave (Evar, EV-30, EQUIMLAB, Mexico City, Mexico) and cooling to below 50 °C. The extract concentrations used were 500, 1000, 2000, 4000, and 8000 mg L^−1^. To perform the agar dilution method, each extract was resuspended in its respective solvent (2 mL), and 0.1% (*v*/*v*) Tween 20 was added to facilitate homogenization in the medium. This was specifically necessary for the hexane extract because preliminary tests identified difficulties in incorporating the lipophilic extract into the hydrophilic culture medium. While the methanolic extract is miscible in water, the surfactant was used to achieve a homogeneous mixture as the concentration of the extract incorporated into the Potato Dextrose Agar medium increased. Six controls were also prepared, as described below: PDA only (control 1), which was prepared according to the manufacturer’s recommendations (39 g L^−1^ of PDA) diluted in distilled water; PDA + solvent (control 2) with 2 mL of solvent and the total volume of PDA medium used for three replicates per strain; PDA + Tween 20 (control 3), with 20 µL of Tween 20 per replicate incorporated into the PDA medium; PDA + Captan^®^ commercial fungicide. To determine the effective concentration of the commercial fungicide Captan^®^, a stock solution (30 mL) was prepared from the manufacturer’s recommended dosage (3 kg ha^−1^), equivalent to 3 × 10^4^ mg L^−1^, considering an application volume of 100 L ha^−1^. From this solution, 1:10, 1:100, and 1:1000 dilutions were prepared, corresponding to concentrations of 3.0, 0.3, and 0.03 mg L^−1^, respectively. To maintain the desired final concentration of the fungicide in the medium, a constant volume of 2 mL of each dilution was added to 18 mL of PDA medium, obtaining a total volume of 20 mL per Petri dish (90 mm). Since the final volume in each dish represents a dilution factor of 0.1 (2/20), the added solutions were prepared 10 times more concentrated than the target final concentration, thus ensuring that the effective concentration of Captan^®^ in the medium exactly corresponded to the 1:10 (control 4), 1:100 (control 5), and 1:1000 (control 6) treatments. The medium was homogenized before solidification to ensure uniform distribution of fungicide. The homogenized agar was poured into Petri dishes with the extract and allowed to solidify. Petri dishes were inoculated by placing a 5 mm plug of each fungus in the center of each plate. The strains were grown in mycelial culture for 12 days. Assessments of the different concentrations were carried out in triplicate, and the inoculated Petri dishes were incubated at 23 ± 2 °C. The efficacy with which each extract treated the fungi was evaluated by measuring the inhibition of mycelial growth (in mm), the diameter of which was measured using a digital Vernier caliper (CALDI-6MP, Truper, Mexico). Measurements were taken every 24 h for 7 days. The percentage of inhibition of mycelial growth was determined using control 1 as a reference according to the method described in [30]:

(1) $$\%\;growth\;=\;\left[\frac{p_{h}}{p_{b}}\right]\times 100\%$$

(2) % GI=100−% growth

where $p_{h}$ is the fungal growth (mm), $p_{b}$ is the fungal growth diameter of the corresponding negative control in each replicate; and % GI is the percentage of growth inhibition.

### 2.5. Statistical Analysis

Experimental trials on antifungal activity were conducted using a completely randomized experimental design. For each fungus, fourteen treatments (two solvents at five different concentrations) and four controls were evaluated. Three replicates were performed per treatment. A multivariate analysis of variance with repeated measures was performed alongside the Hotelling test (α = 0.05). The statistical procedures used Infostat 2020 software, and probit analyses calculated the EC_50_ and EC_90_ values of the extracts using the statistical software package SAS-PC (version 9.1.3) for Windows.

## 3. Results

### 3.1. GC-MS Analysis

The identification of metabolites in the obtained extracts allowed us to recognize various molecules with previously documented biological activity in the scientific literature. Some of these compounds have demonstrated antifungal, antioxidant, antibacterial, and other bioactive functions in different experimental models. The identified compounds are described below, as well as the activities attributed to them in previous studies.

#### 3.1.1. Hexanic Extract

**Figure 2:**
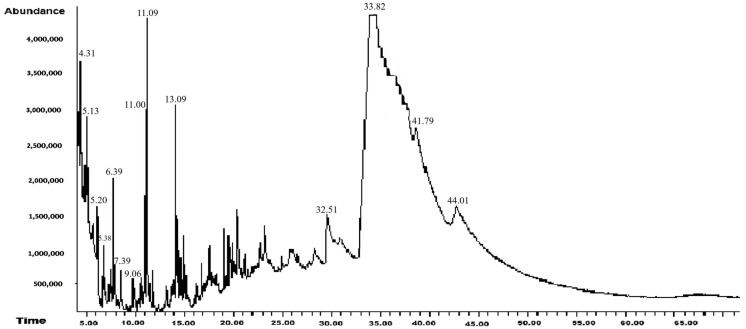
Chromatogram of the hexane extract of *Argemone mexicana* L.

The results of the GC-MS analysis for the hexanic extract derived from leaves of *A. mexicana* are shown in Table 1 and Figure 2. In this extract, 14 compounds were identified, 56% of which were reported to have antibacterial, antifungal, and antiviral activity against pathogenic microorganisms. Only one compound that was previously reported in another study, 1,2,4-Triazol-4-amine, N-(2-thienylmethyl)-, showed antifungal activity against a phytopathogenic fungus. The compounds reported for the first time are N-Methyl-2-isopropoxycarbonylazetidine; Methanesulfinyl fluoride, trifluoro-; and dl-Methionine, N-[ (4-1) methylphenyl) sulfonyl]-.

#### 3.1.2. Methanolic Extract

**Figure 3:**
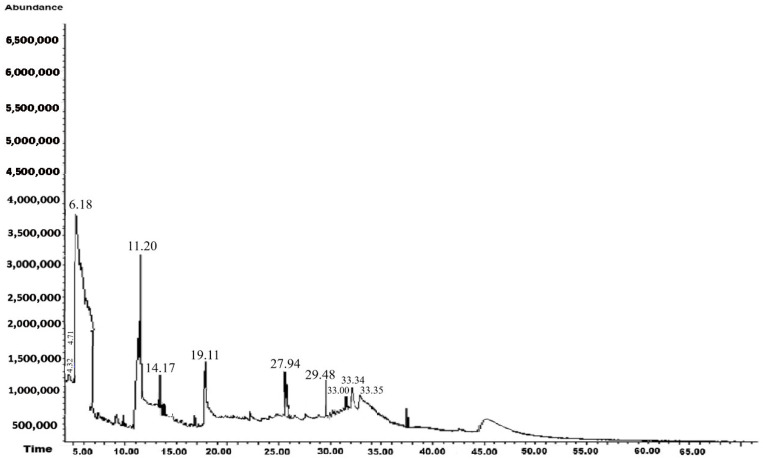
Chromatogram of the methanol extract of *Argemone mexicana* L.

The GC-MS results for the methanolic extract of *A. mexicana* L. are shown in Table 2 and Figure 3. In this extract, 11 compounds were identified, 50% of which exhibited antifungal activity. These were the most abundant compounds in the extract, with Benzene, 1.3-bis(1.1-dimethylethyl)-, being the most common. It is important to mention that antifungal activities of *A. mexicana* extracts against pathogenic fungi have been previously reported; however, this study is the first to identify antifungal activity against *M. fructicola*, *C. gloesoporioides*, *F. oxysporum*, and *S. sclerotinia*. 

### 3.2. In Vitro Evaluation of the Extracts Against Phytopathogenic Fungi

**Figure 4:**
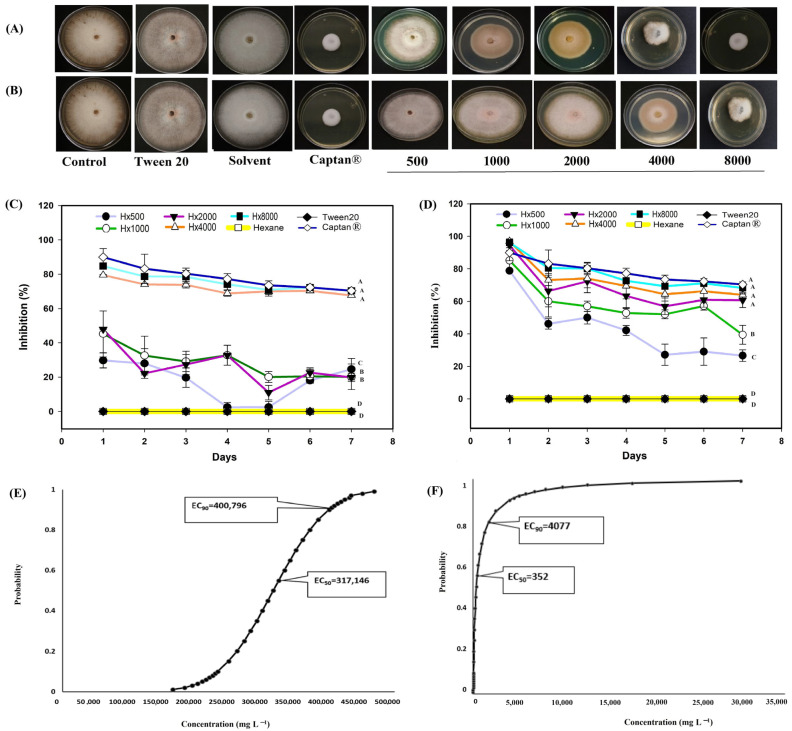
Antifungal effect of *Argemone mexicana* L. extracts against *Monilinia fructicola* after 7 days: (**A**) hexanic extract; (**B**) methanolic extract; (**C**) fungal growth inhibition (%) of the hexanic extract; (**D**) fungal growth inhibition (%) of the methanolic extract; (**E**) Probit analysis of the hexanic extract; and (**F**) Probit analysis of the methanolic extract. Different letters between treatments indicate significant differences according to the Hotelling test (α = 0.05). N = 3 replicates per treatment. Controls: Negative control = PDA only; Tween 20 = PDA + 0.1% Tween 20 surfactant; Solvent = PDA + hexane or methanol; and Control 6: Captan^®^ = PDA +1:1000 Captan. *A. mexicana* extracts contained PDA, surfactant, and solvent. Effective concentrations (EC_50_ and EC_90_) correspond to the fungicide concentrations required to inhibit 50% and 90% of mycelial growth, respectively, compared with the untreated control. These values represent the biological effect produced by the concentration of the extract estimated through dose–response regression analysis.

*A. mexicana* extracts exhibited antifungal activity against the different phytopathogens, such as *M. fructicola*, considered in this study (Figure 4). The hexanic extract showed the strongest biological activity between 4000 and 8000 mg L^−1^ (Figure 4A). Between these concentrations, the hexanic extract inhibited more than 80% of radial growth on the first day (Figure 4C), and the inhibition remained above 70% throughout the experiment. The methanolic extract (Figure 4B) showed a growth inhibition of more than 60% from 2000 mg L^−1^ on the first day (Figure 4D). The Captan^®^ 1:100 (control 6) treatment maintained antifungal activity >80% and from the fourth day onwards the biological activity remained >70% (Figure 4). Probit analysis showed the mean effective concentration required to inhibit the fungicidal activity by 50% (EC_50_) and 90% (EC_90_), respectively, at the end of the experiments (7 days). The hexanic extract had an EC_50_ of 317,146 mg L^−1^ in a range of 312, 879 to 321,090 mg L^−1^, and an EC_90_ of 400,796 mg L^−1^ in a range of 396,131 to 405,958 mg L^−1^ (Figure 4E). By contrast, the methanolic extract had an EC_50_ of 352 mg L^−1^ in a range of 225 to 510 mg L^−1^ (Figure 4F).

**Figure 5:**
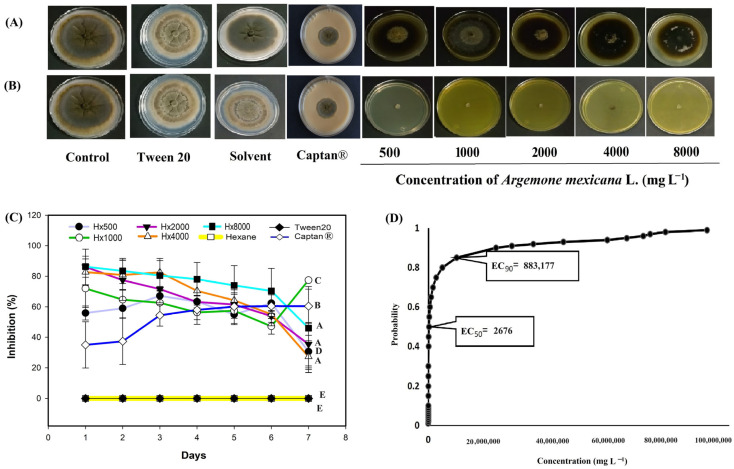
Antifungal effect of *Argemone mexicana* L. extracts against *Colletotrichum gloeosporioides* after 7 days: (**A**) hexanic extract; (**B**) methanolic extract; (**C**) fungal growth inhibition (%) of the hexanic extract; and (**D**) Probit analysis of the hexanic extract. Different letters between treatments indicate significant differences according to the Hotelling test (α = 0.05). N = 3 replicates per treatment. Controls: Negative control = PDA only; Tween 20 = PDA + 0.1% Tween 20 surfactant; Solvent = PDA + hexane or methanol; and Control 6: Captan^®^ = PDA +1:1000 Captan. *A. mexicana* extracts contained PDA, surfactant, and solvent. Effective concentrations (EC_50_ and EC_90_) corresponded to the fungicide concentrations required to inhibit 50% and 90% of mycelial growth, respectively, compared with the untreated control. These values represent the biological effect produced by the concentration of the extract estimated through dose–response regression analysis.

In *C. gloeosporioides* (Figure 5), the hexanic extract inhibited more than 70% growth (Figure 5A) on the six day at a concentration of 8000 mg L^−1^, while the other concentrations exhibited fungistatic activity and kept the percentage of inhibition below 40% throughout the evaluation (Figure 5C). The methanolic extract showed fungicidal activity for the 500 mg L^−1^ treatments (Figure 5B). The Captan^®^ 1:100 (control 6) treatment showed an activity greater than 35% in the first days, from the third day onwards the antifungal activity remained above 50% (Figure 5). Probit analysis for *C. gloeosporioides* (Figure 5D) included only the hexanic extract, as no growth was observed with the methanolic extract. It is possible that the EC_50_ and EC_90_ of this extract are below the 500 mg L^−1^ treatment concentration. The EC_50_ for the hexanic extract was 2676 mg L^−1^, within a range of 1485 and 4409 mg L^−1^.

**Figure 6:**
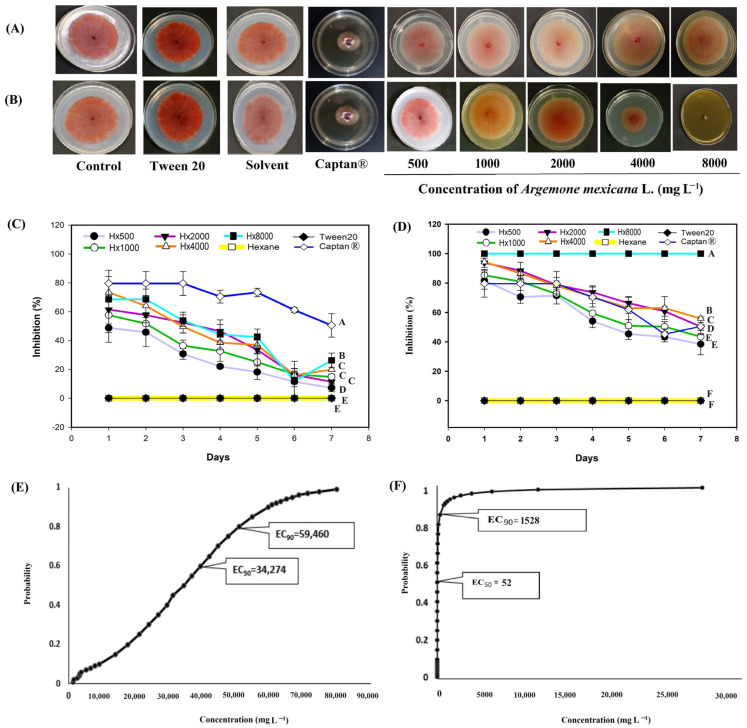
Antifungal effect of *Argemone mexicana* L. extracts against *Fusarium oxysporum* after 7 days: (**A**) hexanic extract; (**B**) methanolic extract; (**C**) fungal growth inhibition (%) of hexanic extract; (**D**) fungal growth inhibition (%) of the methanolic extract; (**E**) Probit analysis of the hexanic extract; and (**F**) Probit analysis of the methanolic extract. Different letters between treatments indicate significant differences according to the Hotelling test (α = 0.05). N = 3 replicates per treatment. Controls: Negative control = PDA only; Tween 20 = PDA + 0.1% Tween 20 surfactant; Solvent = PDA + hexane or methanol; and Control 6: Captan^®^ = PDA +1:1000 Captan. *A. mexicana* extracts contained PDA, surfactant, and solvent. Effective concentrations (EC_50_ and EC_90_) correspond to the fungicide concentrations required to inhibit 50% and 90% of mycelial growth, respectively, compared with the untreated control. These values represent the biological effect produced by the extract concentration estimated through dose–response regression analysis.

For *F. oxysporum* (Figure 6), the hexanic extract only showed >60% inhibition at 8000 mg L^−1^ and during the first two days of evaluation; by day 3, its activity declined, and inhibition at all doses remained below 10% for the rest of the 7-day period (Figure 6C). In contrast, the methanolic extract produced >60% inhibition at all tested concentrations (1000–8000 mg L^−1^) during the first three days (Figure 6D), and at 8000 mg L^−1^, it exhibited fungicidal activity (Figure 6A). Captan^®^ 1:100 treatment inhibited the growth of *F. oxysporum* by over 70% during 5 days of evaluation; at the end of the evaluation, the antifungal activity remained above 50% (Figure 6). Probit analysis for *F. oxysporum* revealed that the hexanic extract of *A. mexicana* (Figure 6E) had an EC_50_ of 34,274 mg L^−1^, within the range of 31,717 and 36,444 mg L^−1^. The EC_50_ of the methanolic extract (Figure 6F) was 52 mg L^−1^, ranging between 1.40 and 320 mg L^−1^. Since the methanolic extract had the lowest EC_50_ concentration, it could be considered a viable option for the control of *F. oxysporum*.

**Figure 7:**
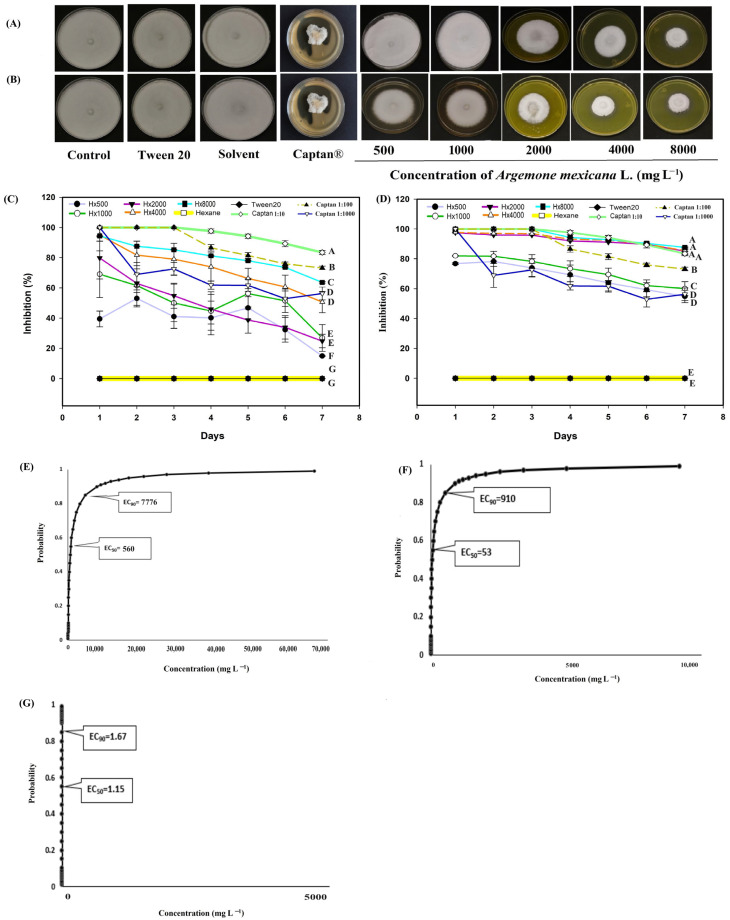
Antifungal effect of *Argemone mexicana* L. extracts against *Sclerotinia sclerotiorum* after 7 days: (**A**) hexanic extract; (**B**) methanolic extract; (**C**) fungal growth inhibition (%) of the hexanic extract; (**D**) fungal growth inhibition (%) of the methanolic extract; (**E**) Probit analysis of the hexanic extract; and (**F**) Probit analysis of the methanolic extract. (**G**) Probit analysis of the Captan^®^. Different letters between treatments indicate significant differences according to the Hotelling test (α = 0.05). N = 3 replicates per treatment. Controls: Negative control = PDA only; Tween 20 = PDA + 0.1% Tween 20 surfactant; Solvent = PDA + hexane or methanol; and Control 6: Captan^®^ = PDA +1:1000 Captan. *A. mexicana* extracts contained PDA, surfactant, and solvent. Effective concentrations (EC_50_ and EC_90_) correspond to the fungicide concentrations required to inhibit 50% and 90% of mycelial growth, respectively, compared with the untreated control. These values represent the biological effect produced by the extract concentration estimated through dose–response regression analysis.

For *S. sclerotiorum* (Figure 7), the hexanic extract was effective at concentrations of 4000 and 8000 mg L^−1^ (Figure 7A,B). At 8000 mg L^−1^, biological activity remained above 60% (Figure 7C), while at concentrations of 4000 and 8000 mg L^−1^, the methanolic extract inhibited growth by more than 90% throughout the evaluation (Figure 7D). In this phytopathogenic fungus the commercial fungicide Captan^®^ presented fungistatic activity with the different concentration gradients 1:10 (control 4) 1:100 (control 5) and 1:1000 (control 6) unlike the rest of the phytopathogenic fungi considered in this experiment, where controls 4 and 5 presented fungicidal activity. For example in *S. sclerotiorum* controls, number 4 and 5 completely inhibited growth throughout the first three days of evaluation, control number 4 (1:10) maintained its efficiency above 80% the rest of the evaluation, while control 5 (1:100) remained above 70% throughout the experiment (Figure 7C,D); With control number 6 (1:1000) the first day presented an inhibition of 100% and throughout the evaluation it had a decline, however the biological efficiency remained above 50% (Figure 7A,B). Probit analysis for *S. sclerotiorum* revealed that the hexane extract had an EC_50_ of 560 mg L^−1^, with a range between 469 and 655 mg L^−1^, and an EC_90_ of 7776 within a range of 6600 and 9384 mg L^−1^ (Figure 7E). The methanolic extract had an EC_50_ of 53 mg L^−1^, and an EC_90_ of 909 mg L^−1^ (Figure 7F). 

Regarding the results of the commercial fungicide Captan^®^, the EC_50_ was 1.19 mg L^−1^ and the EC_90_ was 1.67 mg L^−1^ (Figure 7G). In this probit analysis, as in the methanolic extract, fiducial limits were not obtained because the effective concentration intervals were very narrow. This indicates that the range of concentrations tested was within the zone of maximum sensitivity of the fungus and therefore, the reported values should be interpreted as point estimates within the experimental range. In *M. fructicola*, *C. gloesporioides* and *F. oxysporum*, it was not possible to calculate the EC_50_ and EC_90_ because the gradient interval tested (1:10 and 1:100) inhibited 100% growth, making it impossible to fit a Probit model. However, with the 1:1000 concentration, the observed and calculated inhibition is reported (Figure 4, Figure 5 and Figure 6). Although it was impossible to fit a Probit model for all species, these results clearly reflect the biological behavior of the Captan^®^ fungicide, providing a more accurate picture of its efficacy and a more robust comparison with *A. mexicana* extracts. Although the results indicate that plant extracts have no commercial development value against the four plant pathogens, the plant can be used as green manure to reduce the fungal source of the four pathogens.

## 4. Discussion

The present study demonstrated that *Argemone mexicana* L. extracts exhibit in vitro antifungal activity against the phytopathogenic fungi such as *Monilinia fructicola*, *Colletotrichum gloeosporioides*, *Fusarium oxysporum*, and *Sclerotinia sclerotiorum*. These results confirm our hypothesis that solvent polarity modifies the extract chemical profile and consequent antifungal activity. The methanolic extract was more effective than the hexanic extract, showing complete inhibition of *C. gloeosporioides* and *F. oxysporum* at concentrations ≥4000 mg L^−1^. These findings are consistent with previous reports stating that polar solvents extract a wider diversity of bioactive compounds [18]. The fungistatic behavior of the hexanic extract may be due to its high content of non-polar hydrocarbons and terpenoids that integrate into the fungal membrane but lack reactive functional groups to cause irreversible damage [58]. In contrast, the methanolic extract contained polar phenolic and alkaloid-type compounds capable of hydrogen bonding and oxidative disruption, which likely accounts for its fungicidal effect. The mechanisms of action of plant extracts against phytopathogenic fungi are described below. Although the results are preliminary, they provide valuable evidence that *A. mexicana* contains bioactive secondary metabolites with antifungal potential.

Regarding the behavior of some concentrations of hexane extract against *Monilinia fructicola* (Figure 4C), in *Colletotrichum gloesporioides* (Figure 5C) *Fusarium oxysporum* (Figure 6C) and *Sclerotinia sclerotiorum* (Figure 7C), the inhibition pattern showed a decrease at some points, followed by a notable increase in inhibition and finally showing a slight decrease towards the end of the experiment. This reduction in antifungal efficacy could be related to a delay in the diffusion or solubilization of non-polar compounds present in the hexane extract, which require time to reach the fungal colony and exert their inhibitory action [58,59]. Furthermore, phytopathogenic fungi produce extracellular enzymes and exopolysaccharides that can interact with lipophilic metabolites, temporarily reducing their bioavailability and allowing limited initial mycelial growth [60]. Once these compounds reach an effective concentration in the medium, the inhibition increases significantly, reflecting the accumulated fungistatic activity of the extract [61]. The decrease observed in most bioassays (Figure 4C, Figure 5C, Figure 6C and Figure 7C) after the sixth day could be due to the degradation or volatilization of the active compounds, which is a common phenomenon in extracts rich in terpenoids and hydrocarbons [62].

In Figure 5D, Probit regression analysis yielded EC_50_ = 2,676 mg L^−1^ and EC_90_ = 883,177 mg L^−1^, the latter of which is much higher than the maximum concentration tested (8000 mg L^−1^). This behavior is explained because the dose–response curve presents an asymptotic trend without reaching 100% inhibition within the experimental range [63]. Therefore, the high EC_90_ value should be interpreted as a form of statistical extrapolation and not a biologically applicable parameter. At concentrations above the solubility limit, efficacy did not increase, since the low solubility and volatility of hexane compounds limit their availability in the culture medium [64,65]. Thus, although the Probit model mathematically predicts complete inhibition at extrapolated concentrations, such conditions are not viable or reproducible in practice.

Beyond these concentration–response dynamics, understanding the biochemical basis of the observed effects provides further insight into the antifungal action *of A. mexicana* extracts. Plants’ secondary metabolites are essential components of their immune system, mediating defense responses through complex and dynamic interactions with pathogens. These compounds have evolved over millions of years as part of plants’ adaptation to various biotic stresses, resulting in a chemically diverse repertoire [66]. In order to investigate the mechanisms of action against phytopathogenic fungi, these metabolites were categorized into the following groups: terpenes, phenolic compounds, and nitrogenous compounds [67]. The antifungal activity observed in this study against *M. fructicola*, *C. gloeosporioides*, *F. oxysporum*, and *S. sclerotiorum* (Figure 4, Figure 5, Figure 6 and Figure 7) are attributed to compounds present in hexanic (Table 1 and Figure 2) and methanolic (Table 2 and Figure 3) extracts of *A. mexicana*, including 3,7,11,15-Tetramethyl-2-hexadecen-1-ol; 7,9-Di-tert-butyl-1-oxaspiro(4,5)deca-6,9-diene-2,8-dione and 7,9-Di-tert-butyl-1-oxaspiro(4,5)deca-6,9-diene-2,8-dione (terpenes); Pentanoic acid, 5-hydroxy-, 2,4-di-t-butylphenyl esters and phenol, 2,2′-methylenebis[6-(1,1-dimethylethyl)-4-ethyl- (phenolic compounds); 2-Propanamine, N-methyl-N-nitroso- and 1,2,4-Triazol-4-amine, N-(2-thienylmethyl)-(nitrogenous compounds) [36,41,47,48,49,50,51,52,53,54,55,56].

Mitochondria are essential organelles responsible for energy production, redox balance, and the regulation of apoptosis by maintaining mitochondrial membrane potential [68,69]. In this study, GC-MS analysis of *Argemone mexicana* extracts revealed the presence of the terpene 3,7,11,15-Tetramethyl-2-hexadecen-1-ol; terpenes are hydrocarbons with hydrophobic properties that allow them to integrate into the lipid bilayer of the cell membrane, increasing membrane permeability. This results in a loss of ions and other molecules essential for cell viability and function [70]. Furthermore, terpene-induced disruption of the plasma membrane has been shown to affect mitochondrial membrane potential, triggering imbalances in proton transport, the disruption of ATP synthesis, and the accumulation of reactive oxygen species, which contribute to mitochondrial damage and cell death [71,72]. These mechanisms are consistent with the antifungal activity observed in this investigation against *M. fructicola*, *C. gloeosporioides*, *F. oxysporum*, and *S. sclerotiorum*, since the inhibition of mycelial growth suggests that *A. mexicana* causes damage in the target fungi. Because mitochondrial disruption could potentially affect non-target organisms, future formulations must evaluate selectivity and potential phytotoxicity. Strategies such as nanoencapsulation or emulsified formulations could minimize environmental persistence while maintaining efficacy [64].

In general, terpenes are among the most studied natural antifungals used in botanical products; for example, thymol and carvacrol have an in vitro EC_50_ of =21.3 mg L^−1^ and =9.1 mg L^−1^ against *Botrytis cinerea*, respectively, and values of = 21–50 mg L^−1^ against *F. oxysporum*, depending on the isolate and bioassay conditions. In comparison, for methanolic extracts of *A. mexicana* (52 mg L^−1^), their EC_50_ was of the same order of magnitude but generally higher than pure monoterpenes [61,73,74,75].

Polar solvents, such as methanol, are known to extract phenolic compounds, such as flavonoids, tannins, saponins, unsaturated sterols, triterpenes, and nitrogen-containing compounds, with greater affinity than hexane [18]. For example, pentanoic acid, 5-hydroxy-, 2,4-di-t-butylphenyl esters, and phenol, 2,2′-methylenebis[6-(1,1-dimethylethyl)-4-ethyl are phenolic compounds known to act against pathogens by targeting three sites: the cell wall, the cell membrane, and the mitochondria [76]. Phenolic compounds, including flavonoids, tend to penetrate the hydrophobic core or the lipid bilayer interface, altering its fluidity, permeability, and integrity. These modifications cause the loss of ions (K^+^, H^+^), metabolites, and essential compounds, and disrupt membrane potential and the sodium–potassium pump. Additionally, chelation of divalent cations induces intracellular hyperacidification, triggering alterations in plasma membranes [77,78]. Regarding the inhibition of the mitochondrial electron transport chain (ETC), this leads to a decrease in membrane potential. This inhibition generally occurs in the ETC by inhibiting proton pumps, which reduces ATP synthesis and, therefore, cell death. Finally, the mechanism of action on cell walls suggests that flavonoids inhibit the synthesis of β-glucans and chitin, causing their deformation [79]. Similarly, commercially used chemical fungicides also act on critical cellular sites, albeit through more specific and targeted mechanisms. For example, triazoles such as tebuconazole or propiconazole interfere with ergosterol biosynthesis by inhibiting the enzyme 14α-demethylase (CYP51), which is essential for fungal membrane formation. Synthetic fungicides act on specific enzyme targets, while extracts containing phenolic compounds exert a multisite effect that limits the emergence of resistance [80].

The nitrogen-containing compounds 2-Propanamine, N-methyl-N-nitroso- and 1,2,4-Triazol-4-amine, N-(2-thienylmethyl)- can interfere with the translocation of protons across fungal cell membranes [81]. These compounds act as uncouplers by disrupting the proton gradient required for oxidative phosphorylation and altering the permeability of the mitochondrial membrane, preventing the coupling of the electron transport chain with ATP synthesis [82]. The disruption of adenosine diphosphate (ADP) to ATP phosphorylation directly affects the primary energy metabolism of the fungus and leads to a significant decrease in cellular energy production [83]. In addition, nitrogen compounds inhibit the synthesis of DNA, RNA, proteins, and polysaccharides in phytopathogenic fungi. These compounds bind to enzymes and other important components involved in DNA replication and RNA transcription, preventing the production of nucleic acids [84]. These mechanisms may help explain the antifungal activity observed in this study against *M. fructicola* (Figure 4) and *C. gloeosporioides* (Figure 5), as well as *F. oxysporum* (Figure 6) and *S. sclerotiorum* (Figure 7). For instance, fluytosine (5-fluorocytosine) acts as an analog of cytosine, and when converted to 5-fluorouracil inside the fungal cell, it inhibits thymidylate synthase, blocking DNA and RNA synthesis [85]. Similarly, toyocamycin, a nucleoside analog of adenosine, is misincorporated into RNA and disrupts ribosomal RNA processing, affecting both transcription and translation [86]. Lactimidomycin directly interferes with fungal ribosomes by preventing peptide elongation during protein synthesis [87]. Furthermore, neothramycin, a nitrogen-containing antibiotic, binds to the minor groove of DNA (specifically to guanine residues), thereby blocking the action of DNA and RNA polymerases [88]. Finally, α-amanitin, a type of nitrogen-containing cyclic peptide, selectively inhibits RNA polymerase II via a bridge, thereby halting transcription in the fungal cell [89]. These compounds represent specific molecular targets that compromise fungal cell viability by interfering with its fundamental biosynthetic processes. In particular, these reasons could explain the antifungal activity of *Argemone* extracts against the phytopathogenic fungi investigated in this study (Figure 4, Figure 5, Figure 6 and Figure 7). 

Nitrogen compounds can also interfere with ribosomes and other elements of the translation apparatus and inhibit protein synthesis. This inhibition leads to the disruption of fungal growth and reproduction processes, as proteins and polysaccharides are important structural and functional components in fungal cells [90]. This explains the growth inhibition observed in this study (Figure 4, Figure 5, Figure 6 and Figure 7). While these mechanisms are supported by correlations with previous literature, their confirmation requires genetic or histological studies, including the silencing of genes involved in ergosterol biosynthesis, visualization of mitochondrial damage, and targeted editing using CRISPR–Cas9.

According to the findings of Mitidieri et al. [91], commercial lemon essential oil (*Citrus limon* L.) inhibited the growth of *M. fructicola* by more than 80%. Álvarez-García [92] found that extracts of *Melaleuca alternifolia* Cheel inhibited the growth of *M. fructicola* by more than 40% after three days (Maiden and Betche). These results are consistent with the present study (Figure 4C). De Rodríguez [25] tested the hexanic extract of *Lippia graveolens* Kunth against *C. gloeosporioides* and reported fungistatic activity from 2000 mg L^−1^, which is four times higher than the minimum concentration tested in this study. Feng et al. [93] found that extracts of *Camptotheca acuminata* Decne inhibited more than 50% of growth for *Colletotrichum* at some evaluation points, consistent with the findings of the present study (Figure 5C). Hernández-Soto et al. [30] evaluated the methanolic extract of a plant from the same family, *Argemone ochroleuca*, against *C. gloeosporioides*. They reported growth inhibition of 77% at a concentration of 5000 mg L^−1^. In the present study, 100% inhibition was found at a concentration of 500 mg L^−1^, which is ten times lower than that used in [30] (Figure 5B). Jeewon et al. [94] reported fungicidal activity of *Syzygium aromaticum* (L.) Merr & L.M.Perry methanolic extracts on *F. oxysporum* at a concentration of 9000 mg L^−1^ over a period of seven days, which is 1.12 times higher than the highest concentration considered in the present study. Kordali et al. [95] investigated the activity of hexanic extracts from *Achillea gypsicola* L. and *Achillea biebersteinii* L. against *F. oxysporum* and reported less than 30% growth inhibition throughout the study, similar to the effect reported in the present study (Figure 6C). Saha et al. [96] reported an EC_50_ between 642 and 4915 mg L^−1^ for the hexanic extract of *Tagetes minuta* L. leaves, which is also similar to the values reported in this study (Figure 6E). Pansera et al. [97] evaluated the hexanic extracts of *Boswellia sacra* Flueck. against *S. sclerotiorum* and reported an inhibition of 80%. Tortelli et al. [98] evaluated the extract of *Diaporthe infecunda* R.R.Gomes, Glienke & Crous against the same pathogen, *S. sclerotiorum*, and reported an inhibition of 83%. Both reports are in agreement with the results of the present study (Figure 7D). Onaran et al. [99] tested the methanolic extract of *Rosa canina* L. at a concentration of 1,000 mg L^−1^ and found an inhibition of 40%, which is 0.5 times lower than the values obtained in the present study with the same concentration. Finally, for probit analysis, the literature was reviewed and EC_50_ values for Captan^®^ against *Fusarium oxysporum* of 19.14 mg L^−1^ [100] with intervals between 2.79 and 35.75 mg L^−1^, and 978 mg L^−1^ against *Colletotrichum capsica* [101] were found, without detailed fiducial intervals, the reported concentrations are 16 and 800 times, respectively, above those reported in this research, which suggests a greater sensitivity of the strains studied in this work to Captan^®^ under controlled in vitro conditions.

Differences in fungal susceptibility can be explained by variations in cell wall composition and antioxidant systems. For example, *F. oxysporum* and *S. sclerotiorum* possess thicker chitin–glucan layers and greater tolerance to oxidative stress than *M. fructicola*, reducing their sensitivity to oxidative and membrane-disrupting compounds [102,103,104]. Future research should scale up these results to greenhouse and field trials, considering in vivo efficacy, phytotoxicity, profitability, and effects on non-target organisms (such as pollinators and soil microflora). Although these results justify continued research, the evidence of the antifungal potential of methanolic extracts of *A. mexicana* is preliminary.

## 5. Conclusions

Hexane and methanol extracts of *Argemone mexicana* L. leaves inhibited the mycelial growth of *Monilinia fructicola* and *Colletotrichum gloeosporioides*, two pathogens associated with postharvest diseases, as well as *Fusarium oxysporum* and *Sclerotinia sclerotiorum* fungi, which infect the roots of agriculturally important plants. To our knowledge, this study is the first to report evidence for the in vitro antifungal activity of *A. mexicana* against phytopathogenic fungi. The phytochemical analysis also reports the detection of compounds 2-propenoic acid, 2-methyl-, 1,2-ethanediyl ester; 1,1,3,3-tetramethyl-1,3-disilaphenalane; and cyclohexasiloxane, dodecamethyl- in *A. mexicana* extracts. Although these findings are preliminary and limited to laboratory conditions, they suggest that the compounds present in *A. mexicana* may have potential as natural antifungal agents. Further studies, including in vivo efficacy assays, phytotoxicity tests, and environmental safety assessments, are recommended before considering their application in biofungicide formulations or agricultural disease control strategies.

## Figures and Tables

**Table 1:** Chemical compounds identified in the hexane extracts of *Argemone mexicana* L.

RT (min)	Name of the Compound	Molecular Formula	MW	Area (%)	Previous Reports	Reference
4.31	Butanoic acid, 4-hydroxy-	C_4_H_8_O_3_	104	4.6	Suppressor of the central nervous system	[31]
5.13	Phthalan	C_8_H_8_O	120	6.8	Antidepressant, antioxidant, antifungal, antibacterial, antitumor, and anti-inflammatory properties	[32]
5.20	2,5-Dimethyl-4-hydroxy-3(2H)-furanone	C_6_H_8_O_3_	128	5.8	Aromatic compound that contributes to the flavor of tomatoes	[33]
5.38	2-Hydroxy-gamma-butyrolactone	C_4_H_6_O_3_	102	5.5	Antithyroid and antiviral activity	[34]
6.39	4H-Pyran-4-one, 2,3-dihydro-3,5-dihydroxy-6-methyl-	C_6_H_8_O_4_	144	11.6	Antioxidant, antibacterial, and antidiabetic activity	[35]
7.39	2-Propanamine, N-methyl-N-nitroso-	C_4_H_10_N_2_O	102	2.2	Antibacterial activity against *E. coli*	[36]
9.06	1-(3,6,6-Trimethyl-1,6,7,7a-tetrahydrocyclopenta[c]pyran-1-yl)ethanone	C_13_H_18_O_2_	206	2.0	Insecticidal activity	[37]
11.00	2,4,6-Tris(1,1-dimethylethyl)-4-methylcyclohexa-2,5-dien-1-one	C_19_H_32_O	276	4.7	Insecticidal activity	[38]
11.09	4-(2,6,6-Trimethylcyclohexa-1,3-dienyl)but-3-en-2-one	C_13_H_18_O	190	13.1	Antifungal, antibacterial, antioxidant, anticancer, antispasmodic, hypotensive, and vasorelaxant activity	[39]
13.09	Megastigmatrienone	C_13_H_18_O	190	11.2	Herbicidal activity against *Echinochloa crus-galli* and *Bidens pilosa* and is a biostimulant in rice (*Oryza sativa* L.) cultivation	[40]
32.51	N-Methyl-2-isopropoxycarbonylazetidine	C_8_H_15_NO_2_	157	1.2		
33.82	1,2,4-Triazol-4-amine, N-(2-thienylmethyl)-	C_7_H_8_N_4_S	180	29.3	Antifungal activity against *Rhizoctonia solani*	[41]
41.79	Methanesulfinyl fluoride, trifluoro-	CF_4_OS	136	0.6	Reported for the first time	
44.01	dl-Methionine, N-[(4-methylphenyl)sulfonyl]-	C_12_H_17_NO_4_S_2_	303	1.5	Reported for the first time	

RT: Retention time; MW: Molecular weight; These compounds are the result of 12 replicates per sample.

**Table 2:** Chemical compounds identified in the methanolic extract of *Argemone mexicana* L.

RT (min)	Name of the Compound	Molecular Formula	MW	Area (%)	Previous Reports	Reference
4.32	Methylamine	CH_5_N	31	3.37	Detected in Mediterranean plant species	[42]
4.71	Decane, 6-ethyl-2-methyl-	C_13_H_28_	184	1.13	Industrial use as a solvent for varnishes, paints, and inks	[43]
6.18	Benzene, 1,3-bis(1,1-dimethylethyl)-	C_14_H_22_	190	31.35	Antibacterial and antifungal activity and reports of lipid oxidation activity has been detected in plant extracts of species such as *Asparagus racemosus* Willd and *Ixora coccinea* L.	[44,45,46]
11.20	Pentanoic acid, 5-hydroxy-, 2,4-di-t-butylphenyl esters	C_19_H_30_O_3_	306	17.15	Carboxylic acid with larvicidal, antifungal, flavoring, immunostimulant, antidepressant, and hypocholesterolemic properties	[47,48,49]
14.17	3,7,11,15-Tetramethyl-2-hexadecen-1-ol	C_20_H_40_O	296	10.94	Antibacterial, antifungal and pharmacological activity	[50,51,52]
19.11	7,9-Di-tert-butyl-1-oxaspiro(4,5)deca-6,9-diene-2,8-dione	C_17_H_24_O_3_	276	13.43	Medicinal, antifungal, and antibacterial activity	[53,54]
27.94	Phenol, 2,2′-methylenebis[6-(1,1-dimethylethyl)-4-ethyl-	C_25_H_36_O_2_	368	10.51	Antioxidant and antiviral activity	[55,56]
29.48	10-Nonadecanol	C_19_H_40_O	284	7.55	Antioxidant activity and is present in the hexanic extract of garlic (*Allium sativum* L.)	[57]
33.00	2-Octen-4-one, 2-methyl-	C_9_H_16_O	140	1.55	Reported for the first time	
33.34	2-Benzyl-8-methyl-1H-piperidino[4,3-b]indole	C_19_H_20_N_2_	276	1.88	Reported for the first time	
33.45	Isoxazolo[4,3-a]phenazine, 1-phenyl-	C_19_H_11_N_3_O	297	1.14	Reported for the first time	

RT: Retention time; MW: Molecular weight; These compounds are the result of 12 replicates per sample.

## Data Availability

The original contributions presented in this study are included in the article. Further inquiries can be directed to the corresponding author.
